# Consumer Preference and Willingness to Pay for Nutraceuticals to Prevent Cardiovascular Diseases in Thailand: A Discrete Choice Experiment

**DOI:** 10.3390/nu18183083

**Published:** 2026-09-20

**Authors:** Ajaree Rayanakorn, Khachen Kongpakwattana, Warittha Tieosapjaroen, Piyameth Dilokthornsakul

**Affiliations:** 1 Department of Pharmacology, Faculty of Medicine, Chiang Mai University, Chiang Mai 50200, Thailand; ajaree.rayanakorn@cmu.ac.th; 2 Department of Social and Administrative Pharmacy, Faculty of Pharmaceutical Sciences, Chulalongkorn University, Bangkok 10330, Thailand; khachen.k@chula.ac.th; 3 Melbourne Sexual Health Centre, Alfred Health, Melbourne, Carlton VIC 3053, Australia; wtieosapjaroen@mshc.org.au; 4 School of Translational Medicine, Monash University, Carlton VIC 3053, Australia; 5 Center for Medical and Health Technology Assessment (CM-HTA), Department of Pharmaceutical Care, Faculty of Pharmacy, Chiang Mai University, Chiang Mai 50200, Thailand

**Keywords:** preference, nutraceuticals, cardiovascular disease, willingness to pay, WTP

## Abstract

**Objectives:** Cardiovascular diseases (CVDs) represent a major public health challenge in Thailand, but consumer preferences for preventive nutraceuticals remain poorly understood. This study aimed to assess preferences and willingness to pay for nutraceuticals for cardiovascular disease prevention among the Thai population. **Methods:** A discrete choice experiment (DCE) was undertaken with five attributes, including clarity of evidence, effectiveness, safety, cost, and recommended information sources identified by a comprehensive literature review and qualitative consumer interviews. Thai citizens aged 20 years or older with no history of CVD and able to understand Thai were eligible for this survey. Data collection was conducted using an online survey distributed via social networks between March and April 2026, and the data were analyzed using random parameters logit and latent class logit (LCL) models. **Results:** A total of validated responses from 732 Thai adults were used. Clarity of evidence was the most influential attribute (relative importance: 38.3%), followed by recommendation source (24.3%), effectiveness (15.2%), safety (14.2%), and cost (7.9%). High-quality scientific evidence and healthcare professional recommendations were strongly preferred, yielding the highest monthly WTP of 2215.94 THB (US$67.06) and 2080.28 THB (US$62.95), respectively. Recommendations from social media influencers were viewed unfavorably. LCL analysis identified three distinct consumer segments as value-conscious (37.1%), evidence-oriented (28.4%), and healthcare professional-oriented (34.4%). **Conclusions:** Thai consumers prioritize scientific evidence and healthcare professional guidance over social media or commercial marketing when choosing cardiovascular nutraceuticals. Effective communication and product labeling strategies should be tailored to these distinct consumer segments to support evidence-based self-care.

## 1. Introduction

Cardiovascular diseases (CVDs), including coronary artery disease, stroke, and hypertension, remain the leading causes of global morbidity and mortality, responsible for an estimated 17.9–20 million deaths annually and accounting for a substantial proportion of worldwide healthcare burden [1]. CVD risk is driven by modifiable factors such as dyslipidemia, hypertension, and unhealthy diets, and preventive strategies have become a public health priority to reduce disease incidence and associated costs [1]. In Thailand, CVDs represent a major public health challenge with high mortality and morbidity rates. Recent data indicate that the age-standardized CVD mortality rate in Thailand is substantial, with over 136,000 deaths attributed to cardiovascular causes in 2021, corresponding to an age-standardized mortality rate of approximately 128 per 100,000 population [2]. Hypertension, a primary risk factor for CVD, affects around one-in-four Thai adults and remains poorly controlled, contributing to a high burden of stroke and ischemic heart disease across the country [2].

Lifestyle and nutritional interventions are integral to CVD prevention, complementing pharmacological therapies. Dietary patterns rich in bioactive nutrients and functional compounds have been associated with reduced CVD risk, and biologically active dietary components, including polyphenols, omega-3 fatty acids, and phytosterols, exhibit antioxidant, anti-inflammatory, lipid-lowering, and endothelial-protective mechanisms that may contribute to cardioprotective effects [3]. Despite ongoing research into their clinical efficacy, the scientific evidence supporting specific nutraceuticals in CVD prevention varies in quality and magnitude, and standardized recommendations remain limited [4].

In parallel with these epidemiological trends, interest in nutritional strategies for CVD prevention has grown, particularly products linked to cardiovascular benefits such as lipid lowering, blood pressure modulation, and anti-inflammatory effects. However, supporting individual nutraceuticals remains variable, and consumer use may persist even when evidence of clinical benefit is limited or inconclusive. Several factors may contribute to this discrepancy, including limited consumer awareness of the available clinical evidence, relatively permissive regulation of nutraceutical marketing, and insufficient access to evidence-based advice from healthcare professionals [5]. For example, clinical studies have reported no cardiovascular benefit from multivitamin supplementation [6], while evidence regarding coenzyme Q10 for primary CVD prevention remains inconsistent [7,8,9,10,11,12,13]. These uncertainties highlight the importance of understanding not only the clinical evidence for nutraceuticals, but also how consumers evaluate such evidence when making purchasing decisions [14].

Consumer choice of nutraceuticals is likely to involve trade-offs among multiple product characteristics, including the strength and clarity of scientific evidence, expected effectiveness, safety, price, and source of information or recommendation [15]. However, relatively few studies have quantified the relative importance consumers assign to these attributes or the trade-offs they are willing to make between them. A previous preference study conducted in Malaysia found that consumers placed substantial importance on evidence supporting the safety and effectiveness of nutraceuticals, suggesting that perceptions of scientific credibility can play an important role in product choice [16]. Nevertheless, existing preference research has largely examined nutraceuticals in general rather than products intended specifically for CVD prevention. Consequently, it remains unclear whether consumers prioritize scientific evidence over other attributes, such as effectiveness, safety, cost, and recommendation source, when considering cardioprotective nutraceuticals, and how much they are willing to pay for improvements in these attributes.

These evidence gaps are particularly relevant in Thailand, where the use of nutraceuticals is widespread, and consumers make choices within a distinct healthcare and consumer market context. A recent survey reported that approximately half of the Thai general population used herbal and dietary supplements, despite having only a moderate level of knowledge regarding their use and potential adverse effects [17]. Consumers may therefore rely on different forms of evidence and advice when evaluating nutraceuticals, while differences in purchasing power may influence the trade-offs they make between perceived benefits, safety, and cost [18]. However, evidence on how Thai consumers value these product attributes and how much they are willing to pay for them remains limited. To our knowledge, no previous study has quantified preferences and willingness to pay (WTP) for nutraceuticals specifically intended for CVD prevention among Thai consumers.

A discrete choice experiment (DCE) provides an appropriate approach to address this gap by quantifying the relative importance of scientific evidence, effectiveness, safety, cost, and sources of recommendation, as well as the trade-offs consumers make among these attributes [19]. Such evidence may help healthcare professionals and policymakers better understand consumer decision-making, inform evidence-based communication and product information, and provide insights for stakeholders involved in the development and provision of nutraceuticals for CVD prevention. Therefore, this study aims to evaluate Thai consumers’ preferences and WTP for key attributes of nutraceuticals intended for CVD prevention.

## 2. Methods

### 2.1. Attribute and Level Identification

A mixed-methods approach was used to identify relevant attributes and their corresponding levels for the DCE. This approach combined a literature review with formative qualitative interviews with consumers, following established good research practices for the ISPOR good research practice guidelines for discrete choice experiments and the DCE User Guide [20,21].

A comprehensive search of the PubMed database was conducted on 22 October 2025. The aim was to identify studies reporting factors that influence consumers’ decisions about nutraceutical use for CVD prevention. The search strategy used the following terms: (“Dietary Supplements”[MeSH] OR “Functional Food”[MeSH] OR “dietary supplement*”[tiab] OR “food supplement*”[tiab] OR nutraceutical*[tiab] OR “functional supplement*”[tiab] OR “functional food”[tiab]) AND (“Cardiovascular Diseases”[MeSH] OR cardiovascular[tiab] OR CVD[tiab]) AND (perception[tiab] OR perceiv*[tiab] OR attitude[tiab] OR behaviour*[tiab] OR behavior*[tiab]) AND (interview*[tiab] OR survey*[tiab] OR questionnaire*[tiab]).

The search returned 44 records. Of these, 3 studies met the inclusion criteria and were selected for detailed review. Thematic synthesis was applied to extract factors influencing consumers’ nutraceutical use. The identified factors were perceived health benefits (including effectiveness and clarity of supporting evidence), safety profile, sources of recommendation, cost, and product characteristics such as dosing frequency and brand credibility.

The literature review findings were then used to inform semi-structured in-depth interviews. Following guidance that formative work for a DCE should be conducted with the population whose preferences are to be elicited [22,23,24], five informants were purposively recruited from the target consumer population: Thai adults aged 20 years or older with no diagnosis of cardiovascular disease. Recruitment sought variation in dietary supplement use, occupational sector, and work-related exposure to health-product information. The panel comprised a community pharmacy owner who both retails and uses dietary supplements, a freelance writer who uses none, a senior human resources officer in the petrochemical industry, a senior legal officer in the media industry, and an administrative and finance officer in a university medical faculty (Supplementary Table S1). Interviews were conducted in Thai by AR, KK and PD from November to December 2025 and lasted approximately 30–60 min each. Participants were asked to evaluate and rank the identified factors according to their perceived importance to consumers’ decisions about nutraceutical use for CVD prevention.

The literature review and expert interview findings were triangulated to select the final attributes. Five attributes with corresponding levels were chosen for inclusion in the DCE (see Table 1). These were clarity of evidence (3 levels: low, moderate, and high clarity), effectiveness (4 levels: reduction in cardiovascular risk by 5%, 10%, 15%, and 20%), safety (3 levels: no side effects, mild side effects, and moderate side effects), cost (4 levels: 250, 500, 1000, and 1500 THB/month), and source of recommendation (4 levels: mass media advertising, social media influencers, family members or friends with prior experience using the product, and healthcare professionals). The attribute levels were designed to reflect clinically meaningful and realistic variations based on the existing literature. The interviews informed which attributes were retained, not the numerical levels. The 5% to 20% range for effectiveness was informed by the magnitude of cardiovascular effects reported for omega-3 fatty acid interventions. In a meta-analysis of 38 randomized trials and 149,051 participants, the pooled relative risk for major adverse cardiovascular events was 0.95, whereas eicosapentaenoic acid monotherapy gave 0.78 [24]. The largest single trial reported a 25% reduction in its primary composite endpoint [22]. As in any discrete choice experiment, these levels define the trade-off space presented to respondents rather than claims about the efficacy of marketed products.

### 2.2. Experimental and Survey Design

The online survey was developed using Qualtrics (Qualtrics, Provo, UT, USA). The DCE questionnaire asked respondents with two hypothetical nutraceutical alternatives “Imagine that you want to use nutraceuticals to prevent cardiovascular disease. Which of the following options do you prefer? You may choose either Option A or Option B. If you do not prefer either option, you may choose Option C (“Neither”).” Before deployment, the questionnaire was reviewed by the study team to ensure clarity, comprehensibility, and face validity.

An optimal D-efficient experimental design was generated using NGENE Software (Version 1.2.1, Choicemetrics, Pty Ltd., Sydney, NSW, Australia). The final design comprised 60 choice tasks, which were divided into 10 blocks of six choice tasks to reduce respondent burden. Consequently, 10 questionnaire versions were created, and each respondent was randomly assigned to complete one version containing six choice tasks. In addition, one validity-check choice task containing a dominant alternative (high-quality scientific evidence, 20% reduction in cardiovascular disease risk, no side effects, healthcare professional recommendation, and a cost of 250 THB/month) was included to assess respondents’ understanding of the choice tasks. Respondents selecting the dominant alternative were considered to have passed the internal validity check. An example of a DCE survey is presented in Table 2.

The minimum sample size was estimated using the formula proposed by Johnson and Orme [19,26]:

*n* ≥ 500*c*/*ta*,

where *n* is the minimum number of respondents, *t* is the number of choice tasks per respondent (6), *a* is the number of alternatives per choice task excluding the opt-out option (2), and *c* is the largest number of levels across all attributes (4). Based on this formula, a minimum sample of 167 respondents was required. Assuming that approximately 20% of responses may be incomplete or unusable, the minimum required sample size was 210 participants.

### 2.3. Study Population and Data Collection

Data was collected between March to April 2026 using an anonymous online questionnaire developed in Qualtrics (Qualtrics, Provo, UT, USA). The survey link was disseminated via social media platforms (e.g., Facebook and LINE) using both a web link and a QR code.

Thai citizens aged 20 years or older were eligible to participate if they (1) had never been diagnosed with cardiovascular disease (CVD), including conditions affecting the heart and blood vessels (e.g., myocardial infarction, heart failure, stroke, cardiac arrhythmia, or valvular heart disease); (2) were able to read and understand Thai; and (3) provided electronic informed consent. Individuals without diagnosed CVD who were taking medications for cardiovascular risk-factor management or primary prevention were eligible to participate and were not excluded on the basis of medication use. Individuals were excluded if they had a diagnosed or self-reported mental health disorder that could impair their ability to understand the study information or provide reliable responses.

Upon accessing the survey, participants were presented with an electronic participant information sheet describing the study objectives, eligibility criteria, and other relevant study information. Participants first completed an eligibility screening questionnaire. Those who met all eligibility criteria were invited to provide electronic informed consent before proceeding with the survey.

The questionnaire comprised two sections: (1) the DCE questionnaire and (2) questions on respondents’ demographic characteristics and nutraceutical use. Participation was voluntary, and respondents could withdraw from the survey at any time without penalty. The survey required approximately 10–15 min to complete. Participants who completed the survey were offered 100 THB (approximately US$3.00) as compensation for their time. Compensation was provided via electronic bank transfer or PromptPay to those who voluntarily chose to receive it and provided their payment details. The study was approved by the Research Ethics Committee, Faculty of Medicine, Chiang Mai University (IRB no.091/2026).

### 2.4. Data Synthesis and Analysis

Only responses from participants who correctly selected the dominant alternative in the internal validity choice task were included in the analyses. After excluding the internal validity choice task, each respondent contributed six choice tasks to the final analyses. The dominant choice task is a commonly used internal validity test in discrete choice experiments to assess whether respondents understand the choice task and are attentive when making their selections. Participants who failed this validity check were excluded from the primary analyses [23,27].

Descriptive statistics were calculated using R version 4.5.0 (R Foundation for Statistical Computing, Vienna, Austria) to summarise participants’ characteristics. Choice data were initially explored using multinomial logit (MNL) models. Because DCE data comprise repeated choices made by the same respondent, random parameters logit (RPL) models were subsequently estimated to account for the panel structure of the data and preference heterogeneity. Model selection was based on statistical fit, with comparisons using the log-likelihood (LL) and Akaike Information Criterion per observation (AIC/N).

To investigate observed preference heterogeneity, interaction terms between attribute levels and respondents’ characteristics, including age (≥40 years), sex, and healthcare professional status were incorporated into the RPL models. All parameters were initially specified as random with a normal distribution. Parameters exhibiting little or no significant preference heterogeneity were subsequently specified as fixed to improve model fit. All attribute levels were effects coded [28]. The final RPL model was estimated using 1000 Halton draws to ensure stable simulation of the likelihood function.

Marginal willingness-to-pay (WTP) estimates were obtained by re-estimating the final RPL model with cost specified as a continuous variable while retaining the original coding for all other attributes. WTP was calculated as the negative ratio of the attribute coefficient to the cost coefficient [29,30]. Standard errors and 95% confidence intervals were estimated using the delta method based on the variance–covariance matrix of the final RPL model.

Marginal willingness-to-pay (WTP) = −(β_attribute_/β_cost_)

To identify subgroups of respondents with distinct preference patterns, latent class logit (LCL) models were estimated. Models comprising two, three, and four latent classes were sequentially evaluated. The optimal model was selected based on a combination of statistical fit, model parsimony, and interpretability. Model fit was assessed using the log-likelihood (LL), McFadden’s pseudo R^2^, and the Akaike Information Criterion per observation (AIC/N), with higher LL and pseudo R^2^ values and lower AIC/N values indicating better model fit. Class sizes were also considered to ensure that each latent class represented a meaningful proportion of respondents. All choice models were estimated using NLOGIT 6 (Version 6.0; Econometric Software Inc., Plainview, NY, USA).

## 3. Results

### 3.1. Demographic Characteristics

Of the 893 respondents who completed the survey, 158 were excluded because they failed the internal validity check by not selecting the dominant alternative in the validity-check choice task. The excluded respondents were older (mean 41.1 vs. 37.2 years, *p* < 0.001), less likely to hold a bachelor’s degree or higher (46.2% vs. 78.7%, *p* < 0.001), more likely to earn under 15,000 THB per month (44.9% vs. 16.5%, *p* < 0.001), less likely to be healthcare professionals (25.9% vs. 39.2%, *p* = 0.002), and considerably less likely to use dietary supplements (11.4% current users vs. 35.1%, *p* < 0.001; 59.5% never users vs. 30.2%, *p* < 0.001). They were also more likely to report hypertension (22.2% vs. 12.0%, *p* < 0.001). The groups did not differ by sex (*p* = 0.493) or region (*p* = 0.543) (Supplementary Table S2).

A further three respondents were excluded because they provided no sociodemographic data. This resulted in an analytic sample of 732 respondents (see Table 3). The mean age was 37.2 years (SD 11.1). Most respondents were female (*n* = 527, 72.0%), and most held a bachelor’s degree or above (*n* = 576, 78.7%). The largest income group reported an average monthly household income of 15,000 to 29,999 THB (*n* = 336, 45.9%), followed by 30,000 to 49,999 THB (*n* = 142, 19.4%) and less than 15,000 THB (*n* = 121, 16.5%). For region of residence, 563 respondents (76.9%) were from the Northern region and 73 (10.0%) were from Bangkok and surrounding areas (Greater Bangkok). About two-fifths of respondents (*n* = 287, 39.2%) reported an occupation related to healthcare or medicine.

For general supplement use, 257 respondents (35.1%) currently used dietary supplements, 254 (34.7%) had used them in the past, and 221 (30.2%) had never used them. For cardiovascular supplement use, fish oil or omega-3 was most commonly reported (*n* = 291, 39.8%), followed by coenzyme Q10 (*n* = 89, 12.2%), other supplements (*n* = 84, 11.5%), and garlic extract (*n* = 53, 7.2%). Nonetheless, almost half of the respondents (*n* = 365, 49.9%) reported no use of supplements for cardiovascular health.

For cardiovascular risk factors, 335 respondents (45.8%) reported none. Among those with at least one risk factor, dyslipidemia was the most common (*n* = 204, 27.9%), followed by overweight or obesity (*n* = 165, 22.5%), family history of cardiovascular disease (*n* = 106, 14.5%), hypertension (*n* = 88, 12.0%), diabetes mellitus (*n* = 42, 5.7%), and smoking (*n* = 35, 4.8%). Most respondents were not taking medications to prevent cardiovascular disease (*n* = 626, 85.5%). Of the remainder, 97 (13.3%) were taking such medications and 9 (1.2%) were unsure.

### 3.2. Preferences and Relative Importance of Nutraceutical Preference to Prevent Cardiovascular Disease

The random parameters logit (RPL) model demonstrated a good model fit (log-likelihood = −3414.61; AIC/N = 1.568), indicating significant preference heterogeneity among respondents (see Table 4).

Among the five attributes, clarity of evidence was the most influential factor affecting respondents’ preferences (relative importance: 38.3%), followed by source of recommendation (24.3%), effectiveness (15.2%), safety (14.2%), and cost (7.9%).

Respondents strongly preferred nutraceuticals supported by high-quality scientific evidence (β = 1.56, *p* < 0.01), followed by those supported by moderate-quality evidence (β = 0.60, *p* < 0.01), whereas products supported by low-quality evidence were least preferred (β = −2.16, *p* < 0.01).

For source of recommendation, recommendations from healthcare professionals were most preferred (β = 1.50, *p* < 0.01), followed by recommendations from family members or friends with prior experience using the product (β = 0.20, *p* < 0.05). In contrast, recommendations from social media influencers (β = −0.84, *p* < 0.01) and mass media advertising (β = −0.86, *p* < 0.01) were significantly less preferred.

Regarding effectiveness, respondents preferred nutraceuticals providing greater reductions in cardiovascular disease risk. Products associated with a 20% risk reduction (β = 0.68, *p* < 0.01) and a 15% risk reduction (β = 0.23, *p* < 0.01) were preferred over those providing smaller reductions, whereas a 5% risk reduction was least preferred (β = −0.80, *p* < 0.01). A 10% risk reduction did not significantly influence preferences. Preferences increased monotonically across the four effectiveness levels (β = −0.80, −0.11, 0.23, and 0.68 for 5%, 10%, 15%, and 20% risk reduction, respectively), consistent with expectations of theoretical validity [23].

For safety, respondents preferred nutraceuticals with no side effects (β = 0.64, *p* < 0.01), whereas products associated with moderate side effects were significantly less preferred (β = −0.74, *p* < 0.01). Mild side effects did not significantly affect preferences.

Although cost was the least influential attribute overall, respondents preferred lower-priced nutraceuticals. Products costing 500 Thai baht per month (US$15.13) (β = 0.32, *p* < 0.01) and 250 Thai baht per month (US$7.56) (β = 0.32, *p* < 0.01) were preferred, whereas products costing 1000 Thai baht per month (US$30.26) (β = −0.20, *p* < 0.05) and 1500 Thai baht per month (US$45.39) (β = −0.44, *p* < 0.01) were significantly less preferred.

The opt-out (“Neither”) alternative had a significant negative coefficient (β = −0.97, *p* < 0.01), indicating that respondents generally preferred selecting one of the nutraceutical alternatives rather than choosing neither option.

A sensitivity analysis including 890 respondents, of whom 158 had failed the internal validity check, yielded findings consistent with the primary analysis. Clarity of evidence remained the most influential attribute (relative importance: 37.9%), followed by source of recommendation (23.5%), effectiveness (15.7%), safety (14.8%), and cost (8.1%) (Supplementary Table S3).

### 3.3. Willingness-to-Pay (WTP) for Nutraceutical Attributes for Cardiovascular Disease Prevention

Estimated monthly WTP values are presented in Table 5. Respondents demonstrated the highest WTP for high-quality scientific evidence (2215.94 THB/month (US$67.06); 95% CI 1585.34 to 2846.54), followed by recommendations from healthcare professionals (2080.28 THB/month (US$62.95); 95% CI 1484.83 to 2675.73). Moderate-quality scientific evidence was also highly valued (800.28 THB/month (US$24.22); 95% CI 533.23 to 1067.34).

Respondents were willing to pay 914.81 Thai baht per month (US$27.68) for nutraceuticals associated with a 20% reduction in cardiovascular disease risk, whereas the WTP for a 15% reduction was 378.25 Thai baht per month (US$11.45). In contrast, the WTP for a 10% reduction was not statistically significant.

Regarding safety, respondents were willing to pay 160.93 Thai baht per month (US$4.87) in favor of mild side effects, although this estimate was not statistically significant. Conversely, respondents required compensation equivalent to 1095.04 THB per month (US$33.14) to accept nutraceuticals associated with moderate side effects.

Recommendations from social media influencers were associated with a negative WTP (−1177.93 THB/month (US$−35.65); 95% CI −1557.29 to −798.96), indicating that respondents viewed this recommendation source unfavorably compared with the reference category.

### 3.4. Heterogeneous Preference for Nutraceutical Products

**Figure 1:**
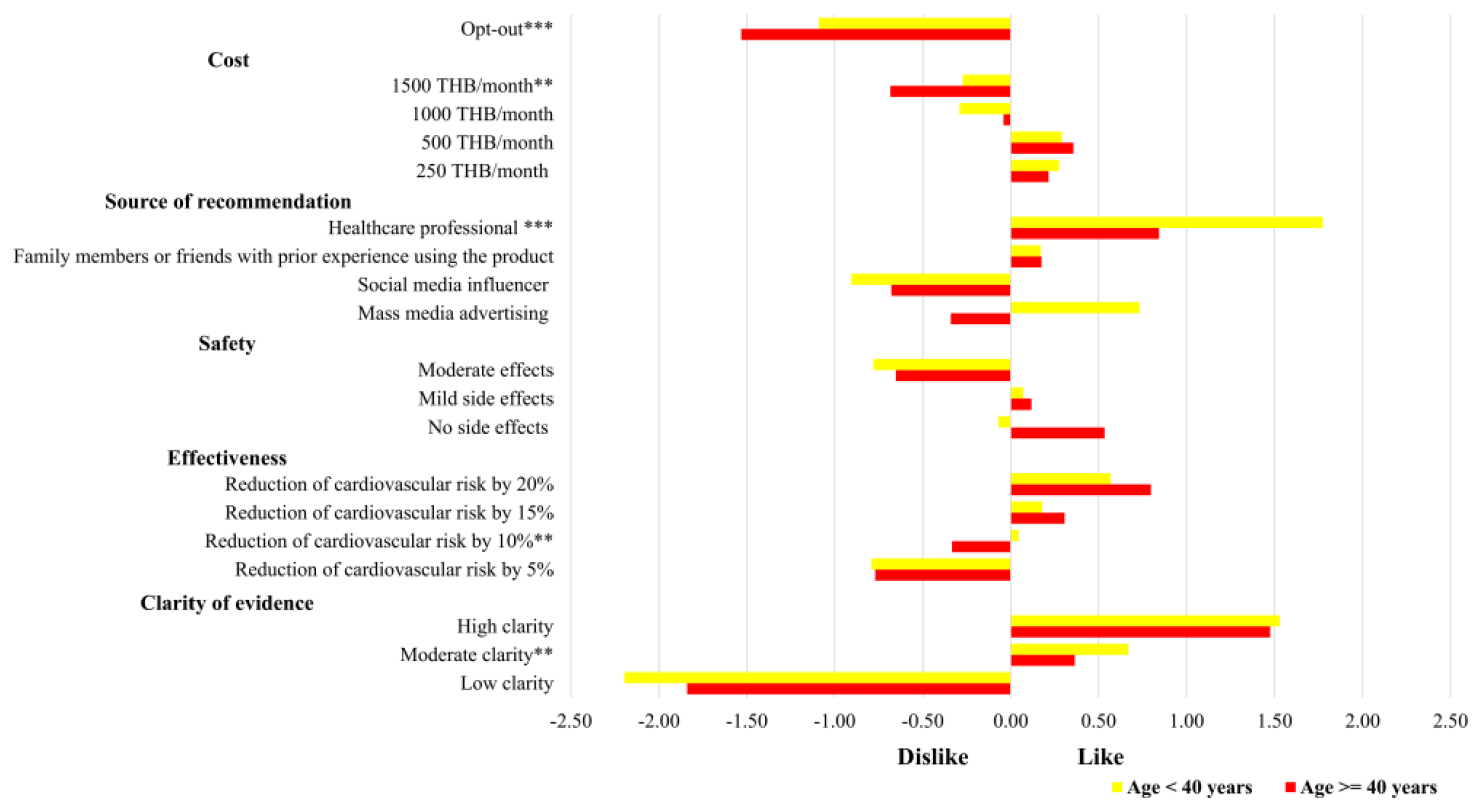
Heterogeneity preference for nutraceuticals to prevent cardiovascular disease according to age group (<40 years vs. ≥40 years). AIC/N = 1.563, Log likelihood function = −3389.58. *** *p*-value < 0.01. ** *p*-value < 0.05.

**Figure 2:**
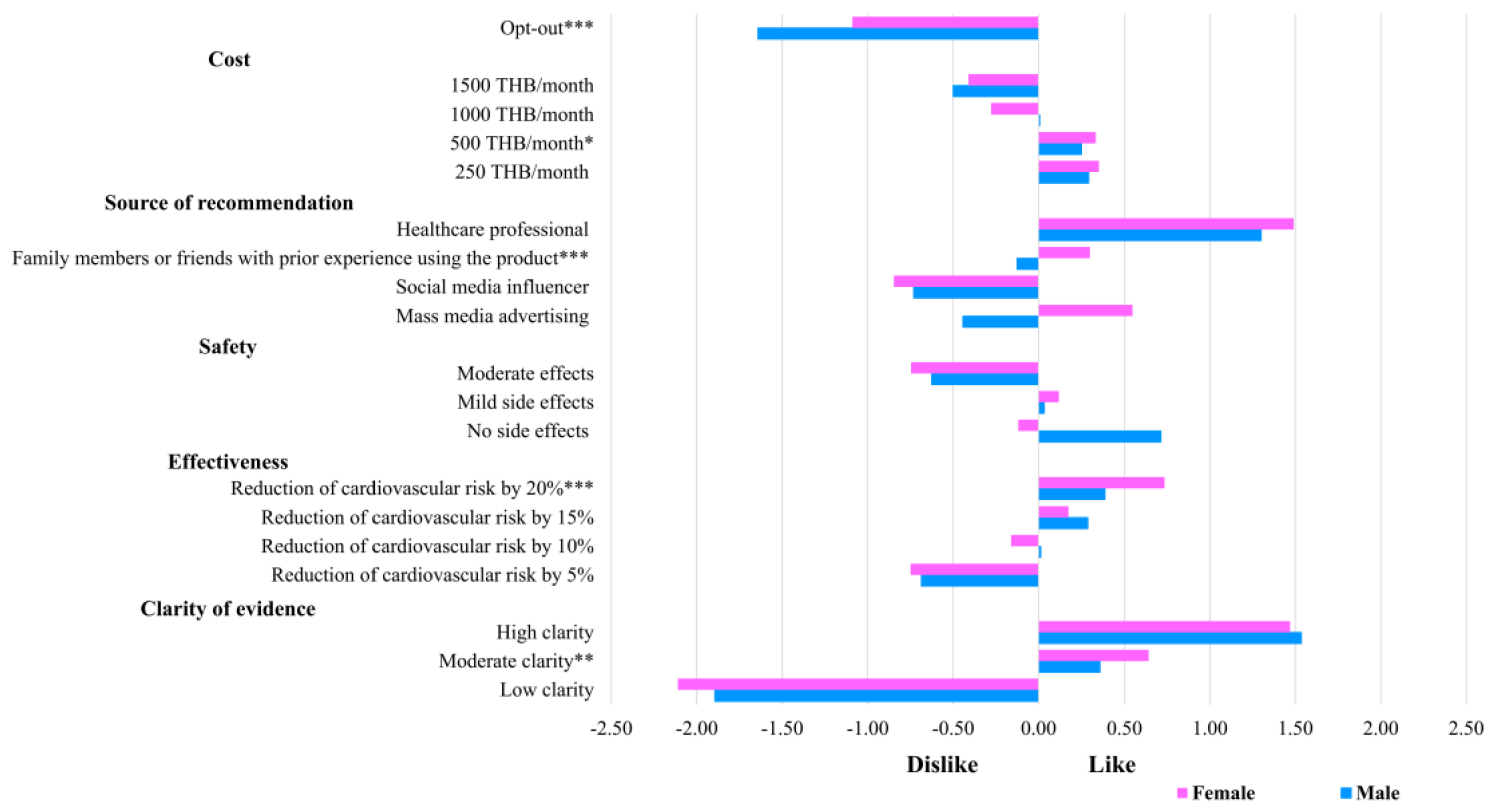
Heterogeneity preference for nutraceuticals to prevent cardiovascular disease among females versus males. AIC/N = 1.571, Log likelihood function = −3407.07. *** *p*-value < 0.01. ** *p*-value < 0.05. * *p*-value < 0.10.

**Figure 3:**
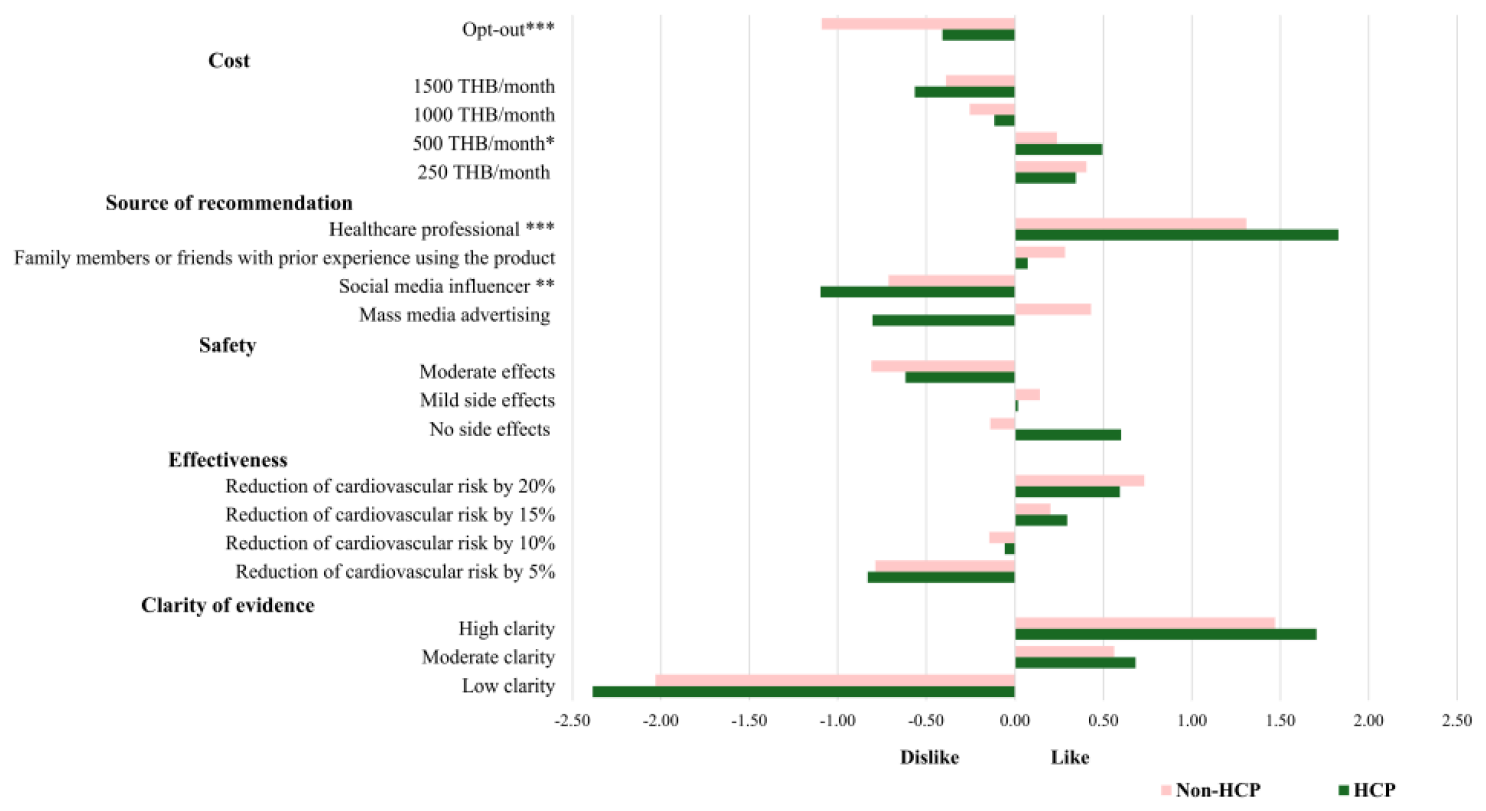
Heterogeneity preference for nutraceuticals to prevent cardiovascular disease according to healthcare provider status. AIC/N = 1.567, Log likelihood function = −3398.71. *** *p*-value < 0.01. ** *p*-value < 0.05. * *p*-value < 0.10.

Interaction analyses indicated modest preference heterogeneity according to age, sex, and healthcare professional (HCP) status (Figure 1, Figure 2 and Figure 3 and Supplementary Table S4 in the Supplemental Materials). The RPL model including interactions with age (≥40 years) demonstrated a modest improvement in model fit compared with the main model (log-likelihood = −3389.58; AIC/N = 1.563). Compared with respondents aged <40 years, those aged ≥40 years placed less importance on moderate-quality scientific evidence, professional healthcare recommendations, and small improvements in effectiveness, while showing greater sensitivity to higher prices (1500 THB or US$45.39/month). They were also less likely to choose the opt-out alternative. No significant age-related differences were observed for safety preferences or larger reductions in cardiovascular disease risk (Figure 1).

Only modest preference heterogeneity according to sex was observed, with a slight improvement in model fit after incorporating sex interactions (McFadden pseudo-R^2^: 0.294 vs. 0.292 for the main RPL model). Compared with females, males placed less importance on a 20% reduction in cardiovascular disease risk and recommendations from family members or friends with prior experience using the product. Males were also less likely to choose the opt-out alternative. No significant sex differences were identified for the remaining attribute levels (Figure 2).

Similarly, incorporating HCP status resulted in only a modest improvement in model fit (McFadden pseudo-R^2^: 0.296 vs. 0.292 for the main RPL model). HCP status did not significantly influence preferences for clarity of evidence, effectiveness, safety, or cost. Compared with non-HCPs, HCPs placed significantly greater importance on recommendation from healthcare professionals (interaction coefficient = 0.52, *p* = 0.004) and exhibited significantly stronger aversion to recommendations from social media influencers (interaction coefficient = −0.38, *p* = 0.021). In addition, HCPs exhibited significantly lower disutility for selecting the opt-out (“Neither”) option than non-HCPs (interaction coefficient = 0.94, *p* < 0.001), indicating a greater propensity to opt-out when the presented nutraceutical alternatives were not sufficiently attractive (Figure 3).

### 3.5. Latent Class Analyses

Latent class analysis identified three distinct preference segments among respondents based on a good balance between model fit, parsimony, and meaningful class sizes (see Supplementary Table S5). The final three-class model had a log-likelihood of −3405.30 and an AIC/N of 1.574. The three latent classes comprised 37.1%, 28.4%, and 34.4% of respondents, respectively (see Table 6).

Class 1 (37.1%), labelled “Value-conscious consumers, placed relatively balanced importance on several attributes. Source of recommendation was the most influential attribute (24.1%), followed by cost (21.7%), clarity of evidence (21.2%), safety (17.0%), and effectiveness (15.9%). Respondents in this class preferred nutraceuticals supported by moderate or high-quality scientific evidence and recommendations from healthcare professionals, while products promoted through mass media advertising or social media influencers were less preferred. Lower-cost nutraceuticals were also favored, indicating greater price sensitivity than the other classes.

Class 2 (28.4%), labelled “Evidence-oriented consumers”, demonstrated the strongest preference for high clarity of evidence, accounting for more than half of overall decision-making (53.0%). This importance substantially exceeded that of effectiveness (16.6%), safety (14.6%), source of recommendation (12.2%), and cost (3.6%). Respondents in this class exhibited the strongest preference for nutraceuticals supported by high-quality scientific evidence, whereas cost had little influence on their choices.

Class 3 (34.4%) labelled “Healthcare professional-oriented consumers, placed the greatest importance on source of recommendation (32.3%), followed by clarity of evidence (31.9%), with safety (13.0%), cost (11.4%), and effectiveness (11.3%) being of comparatively lower importance. Respondents in this class demonstrated the strongest preference for recommendations from healthcare professionals among all latent classes, while recommendations from mass media advertising and social media influencers were least preferred. They also showed a strong preference for nutraceuticals supported by high-quality scientific evidence and were more likely to choose the opt-out alternative than respondents in the other latent classes, suggesting a higher threshold for selecting nutraceutical products.

### 3.6. Respondent Characteristics Associated with Latent Class Membership

Class membership analysis incorporating age ≥ 40 years, HCP status, holding a bachelor’s degree or higher, and never having used nutraceuticals indicated that these respondent characteristics were associated with latent class membership (Supplementary Table S6). Compared with the reference class (Class 3), respondents in Class 1 were more likely to be 40 years or older (β = 0.60, *p* < 0.05), less likely to hold a bachelor’s degree or higher (β = −0.72, *p* < 0.01), and less likely to have never previously used nutraceuticals (β = −0.40, *p* < 0.10). In contrast, none of the respondent characteristics included in the class membership model were significantly distinguished Class 2 from the reference Class 3, suggesting that these two preference segments were not readily differentiated by the demographic and behavioral characteristics evaluated.

## 4. Discussion

This study successfully quantifies consumer preferences and WTP for CVD preventive nutraceuticals among Thai adults. It found that Thai people place the most important value on the scientific credibility of nutraceuticals. Clarity of scientific evidence was the most influential attribute, followed by source of recommendation, while effectiveness and safety had similar intermediate importance and cost was the least influential attribute. The WTP estimates were broadly consistent with the preference weights observed in the random-parameters logit model. Respondents placed the greatest monetary value on products supported by high-quality scientific evidence and recommendations from healthcare professionals, highlighting the importance of evidence credibility and trusted professional advice when selecting nutraceuticals for cardiovascular disease prevention.

One of the key findings of our study was that the clarity of evidence was the most important driver for CVD nutraceutical choice, accounting for 38.3% of the relative importance. The highest estimated WTP was associated with high-quality scientific evidence (THB 2215.94/month). These findings suggest that Thai consumers highly value the credibility of scientific evidence and are willing to pay a substantial premium for nutraceuticals supported by high-quality research.

Another important driver for consumers’ decisions was the recommendation from healthcare professionals. A similarly high WTP was associated with recommendations from healthcare professionals (THB 2080.28/month). Conversely, recommendations from social media influencers were associated with a negative willingness-to-pay, suggesting that this information source may reduce perceived product value. This finding was in line with a previous DCE study in Malaysia which revealed that Malaysian consumers are more likely to buy nutraceutical products with a recommendation from healthcare professionals than someone who has used the product before [16]. Similarly, a study from Croatia revealed that a recommendation by pharmacists could significantly affect consumers’ decisions, even though the magnitude of effect is slightly lower than a recommendation from family and friends [31]. This finding emphasizes the importance of accurate information delivered by healthcare professionals. Thus, healthcare professionals should be careful when providing nutraceutical information for consumers or the public.

Effectiveness and safety had broadly similar influence on respondents’ choices, accounting for 15.2% and 14.2% of relative importance, respectively. Respondents preferred greater reductions in cardiovascular risk and strongly disliked products associated with moderate adverse effects. In particular, the negative preference weight for moderate adverse effects indicates that potential harm is an important consideration in product choice. However, this should not be interpreted as evidence that safety was more important overall than effectiveness. These findings suggest that both expected benefits and potential adverse effects should be communicated clearly and supported by credible evidence when providing information about nutraceuticals for CVD prevention.

A previous systematic review of 76 studies has revealed that the lack of beliefs and knowledge in the effectiveness of nutraceuticals might lead to negative decisions regarding the use of nutraceuticals [15]. Consequently, consumers often depend on healthcare professionals to provide the clinical evidence they prefer regarding nutraceuticals. It is crucial, therefore, that these professionals rely on valid clinical data to offer accurate and clear advice, particularly concerning health claims, given that prior research highlights their limited ability to retrieve reliable evidence on these products.

The latent class findings further suggest that a single communication approach may not adequately address heterogeneous consumer preferences. Value-conscious consumers considered multiple attributes relatively evenly and were more likely to be older and to have previous experience with nutraceuticals, suggesting that communication for this segment may benefit from combining credible evidence and professional recommendations with transparent information on price and product value. Evidence-oriented consumers placed substantially greater importance on the clarity of scientific evidence, suggesting that transparent communication of the quality and strength of supporting evidence may be particularly important for this segment. However, this class was not clearly distinguished from the healthcare professional-oriented class by the demographic and behavioral characteristics examined, indicating that demographic characteristics alone may be insufficient for identifying evidence-oriented consumers. Healthcare professional-oriented consumers placed particularly strong emphasis on recommendations from healthcare professionals and scientific evidence, highlighting the potential role of healthcare professionals in providing credible, evidence-based nutraceutical information. Together, these findings suggest that communication strategies should reflect differences in consumers’ information needs and decision criteria rather than relying solely on demographic segmentation.

Several limitations must be considered when interpreting these findings. First, the sample is characterized by a geographic and demographic imbalance, with 76.9% of respondents residing in the Northern region of Thailand, 72.0% being female, 78.7% holding a bachelor’s degree or higher, and 39.2% of the cohort worked in healthcare or medicine. These characteristics differ from those of the general Thai population and may have influenced the estimated importance of the attributes, particularly the emphasis placed on scientific evidence and cost. Accordingly, the relative-importance estimates should not be interpreted as nationally representative. Preference heterogeneity by sex was modest, suggesting that the over-representation of women was unlikely to materially alter the overall ranking of attributes. Post-stratification weighting to the national regional distribution was not applied, because some regions were represented by very few respondents, which would have produced extreme and unstable weights. Future studies should consider quota-based or probability-based sampling across regions, sex, and socioeconomic groups. Second, recruitment relied on an online convenience sample disseminated through social media platforms, which may have underrepresented older adults and individuals with lower digitally literacy, who may have different preferences.

Third, cost was initially specified as a categorical attribute in the DCE, making direct application conventional WTP potentially problematic. To address this issue, we re-estimated the final RPL model with cost specified as a continuous variable and calculated WTP as the ratio of the attribute coefficient to the negative of the cost coefficient [30]. Nevertheless, the resulting WTP estimates should be interpreted cautiously. In particular, the relatively high values associated with high-quality scientific evidence (THB 2215.94/month) and recommendations from healthcare professionals (THB 2080.28/month) represent monetary valuations of individual attribute levels under the specified model and should not be interpreted as expected market prices or actual monthly expenditures on nutraceuticals. Some estimates exceeded the highest cost level presented in the DCE (THB 1500/month), thereby involving extrapolation beyond the experimentally evaluated cost range. Although a recent survey reported that approximately half of Thai consumers allocated THB 1000–3000 per month to healthcare-related expenses, including vitamins, health products, and dietary supplements [32], these expenditures are not directly comparable with the marginal WTP estimates obtained in our study. In addition, because the DCE involved hypothetical choices without actual financial consequences, hypothetical bias may have led to higher stated WTP than would be observed in real purchasing situations. Actual purchasing behavior may also be constrained by income, household budgets, prevailing product prices, and other market factors.

Fourth, the qualitative interviews used to inform attribute selection involved five members of the target consumer population rather than clinical or nutrition specialists. This approach was consistent with guidance emphasizing the perspective of prospective respondents. Although three of the five attributes were non-clinical, their relevance and salience were established from the consumer perspective alone. Fifth, the effectiveness levels were informed by effect sizes reported for omega-3 fatty acid interventions and should not be interpreted as implying that nutraceuticals marketed in Thailand would achieve a 20% reduction in cardiovascular risk. Sixth, effectiveness was presented as a relative risk reduction without an accompanying baseline risk. Because eligible respondents had no diagnosed CVD and their absolute cardiovascular risk varied according to age, sex, and metabolic risk factors, no single baseline risk would have been appropriate for the entire sample. Respondents could therefore compare the relative ordering of the effectiveness levels but could not translate them directly into absolute risk reductions. Although preferences were monotonic across the four effectiveness levels, indicating that respondents distinguished them in the expected direction, this does not establish that all respondents interpreted the absolute magnitude of risk reduction similarly. Future studies could improve interpretability by presenting a standardized baseline risk or using natural-frequency formats. Finally, as with all DCE, the choices presented were hypothetical. While internal validity checks were utilized to exclude inattentive respondents, actual purchasing decisions in a real-world setting may be influenced by factors not captured in the experiment, including brand loyalty, immediate product availability, and actual budget constraints.

## 5. Conclusions

Thai consumers prioritize scientific evidence and healthcare professional guidance when choosing nutraceuticals for CVD prevention. The identified preference segments suggest that communication should reflect different consumer priorities, emphasizing affordability and overall product value for value-conscious consumers, transparent scientific evidence for evidence-oriented consumers, and evidence-based professional guidance for healthcare professional-oriented consumers. Across segments, credible evidence, safety, affordability, and trusted information sources were important determinants of choice. These findings may inform more transparent and appropriately targeted communication to support informed consumer decision-making.

## Figures and Tables

**Table 1:** Attributes and levels of the discrete choice experiment.

Attributes	Levels
Clarity of evidence	Low clarity * Moderate clarity ** High clarity ***
Effectiveness	Reduction in cardiovascular risk by 5% † Reduction in cardiovascular risk by 10% †† Reduction in cardiovascular risk by 15% ‡ Reduction in cardiovascular risk by 20% ‡‡
Safety	No side effects Mild side effects Moderate effects
Cost	250 THB/month (US$7.56) 500 THB/month (US$15.13) 1000 THB/month (US$30.26) 1500 THB/month (US$45.39)
Source of recommendation	Mass media advertising Social media influencer Family members or friends with prior experience using the product Healthcare professional

* Limited research evidence or unclear study results; ** Supported by some studies, but the number, quality, or consistency of results is limited; *** Supported by several high-quality studies with consistent results; † The relative risk of experiencing a major cardiovascular event (such as a heart attack or stroke) was lowered by 5% compared to those not taking nutraceuticals; †† The relative risk of experiencing a major cardiovascular event (such as a heart attack or stroke) was lowered by 10% compared to those not taking nutraceuticals; ‡ The relative risk of experiencing a major cardiovascular event (such as a heart attack or stroke) was lowered by 15% compared to those not taking nutraceuticals; ‡‡ The relative risk of experiencing a major cardiovascular event (such as a heart attack or stroke) was lowered by 20% compared to those not taking nutraceuticals. The average exchange rate in 2025 was $US1 = 33.0453 THB [25].

**Table 2:** Example of a discrete choice experiment (DCE) choice task presented to participants.

	Option A	Option B	Option C
Clarity of evidence	High clarity	Low clarity	I don’t like options A or B
Effectiveness	Reduction in cardiovascular risk by 20%	Reduction in cardiovascular risk by 10%	
Safety	Moderate effects	Mild side effects	
Cost	500 THB/month (US$15.13)	1000 THB/month (US$30.26)	
Source of recommendation	Family members or friends with prior experience using the product	Healthcare professional	

Note. Participants were instructed as follows: “Imagine that you want to use nutraceuticals to prevent cardiovascular disease. Which of the following options do you prefer? You may choose either Option A or Option B. If you do not prefer either option, you may choose Option C (“Neither”).”.

**Table 3:** Sociodemographic characteristics of the study population (*N* = 732).

Characteristics	Value
Age (years), mean (SD)	37.2 (11.1)
Sex, *n* (%) - Male - Female	205 (28.0%) 527 (72.0%)
Highest level of education, *n* (%) - Primary school or below - Lower secondary (Grade 7–9) - Upper secondary (Grade 10–12)/Vocational certificate - Associate degree/Higher vocational certificate - Bachelor’s degree or above	14 (1.9%) 15 (2.1%) 71 (9.7%) 56 (7.7%) 576 (78.7%)
Average monthly household income, *n* (%) - Less than 15,000 THB - 15,000–29,999 THB - 30,000–49,999 THB - 50,000–74,999 THB - 75,000–99,999 THB - 100,000 THB or more	121 (16.5%) 336 (45.9%) 142 (19.4%) 72 (9.8%) 26 (3.6%) 35 (4.8%)
Region of current residence, *n* (%) - Bangkok and surrounding areas (Greater Bangkok) - Central region - Northern region - Northeastern region - Eastern region - Western region - Southern region	73 (10.0%) 29 (4.0%) 563 (76.9%) 45 (6.2%) 2 (0.3%) 3 (0.4%) 17 (2.3%)
Occupation related to healthcare or medicine (e.g., physician, pharmacist, nurse, physical therapist), *n* (%) - Yes - No	287 (39.2%) 445 (60.8%)
Use of dietary supplements, *n* (%) - Currently using - Previously used, but not currently - Never used	257 (35.1%) 254 (34.7%) 221 (30.2%)
Use of dietary supplements for cardiovascular health *, *n* (%) - Fish oil/Omega-3 - Coenzyme Q10 - Garlic extract - Other - Never used	291 (39.8%) 89 (12.2%) 53 (7.2%) 84 (11.5%) 365 (49.9%)
Cardiovascular risk factors *, *n* (%) - Dyslipidaemia - Hypertension - Diabetes mellitus - Overweight or obesity - Smoking - Family history of cardiovascular disease - No risk factor	204 (27.9%) 88 (12.0%) 42 (5.7%) 165 (22.5%) 35 (4.8%) 106 (14.5%) 335 (45.8%)
Currently taking conventional medications to prevent cardiovascular disease, *n* (%) - Yes - No - Not sure	97 (13.3%) 626 (85.5%) 9 (1.2%)

* The sum of responses may exceed the total number of participants (*n* = 732), as each participant may select more than one option.

**Table 4:** Preferences for nutraceuticals to prevent cardiovascular diseases (*N* = 732), random parameters logit model.

Attribute	Level	Relative Importance (%)	Coefficient	Standard Error	Standard Deviation
Clarity of evidence	Low clarity	38.3	−2.16 ***	0.16	1.51
	Moderate clarity		0.60 ***	0.08	0.80
	High clarity		1.56 ***	0.12	1.28
Source of recommendation Source of recommendation	Mass media advertising	24.3	−0.86 ***	0.11	1.22
	Social media influencer		−0.84 ***	0.09	0.10
	Family members or friends with prior experience using the product		0.20 **	0.08	0.59
	Healthcare professional		1.50 ***	0.12	1.06
Effectiveness	Reduction in cardiovascular risk by 5%	15.2	−0.80 ***	0.10	1.06
	Reduction in cardiovascular risk by 10%		−0.11	0.08	0.43
	Reduction in cardiovascular risk by 15%		0.23 ***	0.08	0.41
	Reduction in cardiovascular risk by 20%		0.68 ***	0.09	0.88
Safety	No side effects	14.2	0.64 ***	0.07	0.82
	Mild side effects		0.10	0.06	0.38
	Moderate effects		−0.74 ***	0.08	0.72
Cost	250 THB/month (US$7.56)	7.9	0.32 ***	0.08	0.91
	500 THB/month (US$15.13)		0.32 ***	0.08	0.18
	1000 THB/month (US$30.26)		−0.20 **	0.08	0.25
	1500 THB/month (US$45.39)		−0.44 ***	0.09	0.86
Opt-out			−0.97 ***	0.16	3.05

Note. All attributes were effects coded. Coefficients and standard errors for the reference levels were derived using the delta method based on the estimated covariance matrix (VARB). Statistical significance was assessed using Wald z-tests derived from the estimated coefficient and standard error. The average exchange rate in 2025 was $US1 = 33.0453 THB [25]. AIC/N = 1.568; Log likelihood function = −3414.61. *** *p*-value < 0.01. ** *p*-value < 0.05.

**Table 5:** Estimated monthly willingness-to-pay (WTP) for nutraceutical attributes for cardiovascular disease prevention.

Attribute	Level	WTP (THB/Month)	Standard Error	95% Confidence Intervals (CIs)
Clarity of evidence	Low clarity	−3016.22 (US$−91.28)	441.26	−3880.30 to −2152.15
	Moderate clarity	800.28 (US$24.22)	136.25	533.23 to 1067.34
	High clarity	2215.94 (US$67.06)	321.74	1585.34 to 2846.54
Source of recommendation	Mass media advertising	−1192.18 (US$−36.08)	227.98	−1639.01 to −745.35
	Social media influencer	−1177.93 (US$−35.64)	193.55	−1557.29 to −798.96
	Family members or friends with prior experience using the product	289.82 (US$8.77)	115.28	63.88 to 515.76
	Healthcare professional	2080.28 (US$62.95)	303.80	1484.83 to 2675.73
Effectiveness	Reduction in cardiovascular risk by 5%	−1158.63 (US$−35.06)	207.40	−1565.13 to −752.12
	Reduction in cardiovascular risk by 10%	−134.43 (US$−4.07)	108.47	−347.04 to 78.17
	Reduction in cardiovascular risk by 15%	378.25 (US$11.45)	123.63	135.95 to 620.56
	Reduction in cardiovascular risk by 20%	914.81 (US$27.68)	167.48	586.54 to 1243.07
Safety	No side effects	934.12 (US$28.27)	154.13	632.02 to 1236.21
	Mild side effects	160.93 (US$4.87)	91.37	−18.17 to 340.02
	Moderate effects	−1095.04 (US$−33.14)	180.11	−1448.05 to −742.04

CIs, confidence intervals; THB, Thai baht; WTP, willingness-to-pay. Note. Standard errors and 95% confidence intervals were estimated using the delta method based on the variance–covariance matrix of the final random-parameters logit model. The average exchange rate in 2025 was $US1 = 33.0453 THB [25].

**Table 6:** Latent class logit model of preferences for nutraceuticals to prevent cardiovascular disease (*N* = 732).

	Class Name	Class 1: Value-Conscious Consumers		Class 2: Evidence-Oriented Consumers		Class 3: Healthcare Professional-Oriented Consumers	
	Size (%)	37.1		28.4		34.4	
Attribute	Level	Coefficient	Standard Error	Coefficient	Standard Error	Coefficient	Standard Error
Clarity of evidence	Low clarity	−0.43 ***	0.07	−2.90 ***	0.39	−1.64 ***	0.14
	Moderate clarity	0.19 ***	0.06	0.74 ***	0.16	0.50 ***	0.09
	High clarity	0.24 ***	0.06	2.16 ***	0.29	1.15 ***	0.11
Effectiveness	Reduction of cardiovascular risk by 5%	−0.23 ***	0.07	−1.00 ***	0.17	−0.61 ***	0.12
	Reduction in cardiovascular risk by 10%	−0.04	0.06	−0.02	0.15	−0.01	0.10
	Reduction in cardiovascular risk by 15%	0.01	0.07	0.43 **	0.17	0.24 **	0.10
	Reduction in cardiovascular risk by 20%	0.26 ***	0.08	0.59 ***	0.15	0.38 ***	0.10
Safety	No side effects	0.24 ***	0.05	0.70 ***	0.14	0.55 ***	0.09
	Mild side effects	0.06	0.05	−0.01	0.12	0.03	0.08
	Moderate effects	−0.29 ***	0.05	−0.69 ***	0.12	−0.59 ***	0.09
Source of recommendation	Mass media advertising	−0.33 ***	0.08	−0.07	0.16	−0.87 ***	0.13
	Social media influencer	−0.29 ***	0.07	−0.52 ***	0.15	−1.06 ***	0.15
	Family members or friends with prior experience using the product	0.19 ***	0.07	−0.07	0.19	0.17	0.11
	Healthcare professional	0.43 ***	0.07	0.65 ***	0.22	1.76 ***	0.12
Cost	250 THB/month (US$7.56)	0.34 ***	0.07	0.11	0.143	0.30 ***	0.10
	500 THB/month (US$15.13)	0.13 **	0.06	−0.07	0.17	0.48 ***	0.11
	1000 THB/month (US$30.26)	−0.14 **	0.07	−0.19	0.17	−0.24 **	0.11
	1500 THB/month (US$45.39)	−0.33 ***	0.07	0.15	0.19	−0.53 ***	0.12
Neither		−2.78 ***	0.25	−0.81 ***	0.26	1.58 ***	0.13
Class membership model	Being an HCP	−0.47 ***	0.22	−0.28	0.24	Reference	
	Age ≥ 40 years	0.60 **	0.23	0.35	0.26	Reference	
	Hold a bachelor’s degree of above	−0.72 ***	0.27	0.19	0.34	Reference	
	Never use nutraceuticals	−0.40 *	0.21	0.32	0.24	Reference	
Importance of attribute (%)							
Clarity of evidence		21.2		53.0		31.9	
Effectiveness		15.9		16.6		11.3	
Safety		17.0		14.6		13.0	
Source of recommendation		24.1		12.2		32.3	
Cost		21.7		3.6		11.4	

Note. All attributes were effects coded. Coefficients and standard errors for the reference levels were derived using the delta method based on the estimated covariance matrix (VARB). Statistical significance was assessed using Wald z-tests derived from the estimated coefficient and standard error. HCP, Healthcare professional. THB, Thai Baht. AIC/N = 1.574; Log likelihood function = −3405.302. *** *p*-value < 0.01. ** *p*-value < 0.05. * *p*-value < 0.10.

## Data Availability

The data and analysis code presented in this study are available from the corresponding author on reasonable request.

## Supplementary Materials

The following supporting information can be downloaded at https://www.mdpi.com/article/10.3390/nu18183083/s1, Table S1. Description of the five panel informants; Table S2. Comparison of excluded and included respondents; Table S3. Sensitivity analysis using random parameters logit model for preferences nutraceuticals to prevent cardiovascular diseases including those who failed the internal validity test (*N* = 890); Table S4. Comparison of parameters, Log-likelihood, McFadden R2 and AIC/N between MNL, Full RPL and Reduced RPL models; Table S5. Comparison of latent class logit (LCL) models between 2, 3, and 4 classes; Table S6. Comparison of 3 classes latent class logit (LCL) model with covariates.
