# Supplementary material for: Consumer Preference and Willingness to Pay for Nutraceuticals to Prevent Cardiovascular Diseases in Thailand: A Discrete Choice Experiment
Source: Nutrients. 2026 Sep 20;18(18):3083. doi: 10.3390/nu18183083 (PMC13610477; doi:10.3390/nu18183083)
Supplement: Supplementary file 1 [file nutrients-18-03083-s001.zip › nutrients-4556684-supplementary.pdf]

Supplementary

Table S1 Description of the five panel informants

ID	Occupation	Sector	Supplement user	Age	Sex
P1	Community pharmacy owner	Retail pharmacy	Yes	42	Female
P2	Freelance writer	Media and publishing	No	66	Male
P3	Senior human resources officer	Petrochemical industry	Yes	41	Female
P4	Senior legal officer	Media and entertainment	Yes	35	Male
P5	Administrative and finance officer	University, medical faculty	Yes	48	Female

Table S2 Comparison of excluded and included respondents

Characteristic	Analytic sample (n = 732)	Excluded (n = 158)	*p-value*
Age, mean (SD), years	37.2 (11.1)	41.1 (12.8)	<0.001
Female, *n* (%)	527 (72.0)	118 (74.7)	0.493
Bachelor's degree or higher, *n* (%)	576 (78.7)	73 (46.2)	<0.001
Income < 15,000 THB/month, *n* (%)	121 (16.5)	71 (44.9)	<0.001
Northern region, *n* (%)	563 (76.9)	124 (78.5)	0.670
Greater Bangkok, *n* (%)	73 (10.0)	10 (6.3)	0.153
Healthcare professional, *n* (%)	287 (39.2)	41 (25.9)	0.002
Current supplement user, *n* (%)	257 (35.1)	18 (11.4)	<0.001
Never used supplements, *n* (%)	221 (30.2)	94 (59.5)	<0.001
Any CVD risk factor, *n* (%)	397 (54.2)	72 (45.6)	0.048
Dyslipidemia, *n* (%)	204 (27.9)	36 (22.8)	0.192
Hypertension, *n* (%)	88 (12.0)	35 (22.2)	<0.001
Diabetes, *n* (%)	42 (5.7)	14 (8.9)	0.143

Note. Chi-square or Fisher's exact test for categorical variables, Welch t-test for age. One excluded respondent had missing sociodemographic data.

Table S3 Sensitivity analysis using random parameters logit model for preferences nutraceuticals to prevent cardiovascular diseases including those who failed the internal validity test (*N*=890)

Attribute	Level	Relative importance (%)	Coefficient	Standard error	Standard deviation
Clarity of evidence	Low clarity	37.9	-1.73***	0.12	1.35
	Moderate clarity		0.46***	0.06	0.72
	High clarity		1.27***	0.09	1.14
Source of recommendation Source of recommendation	Mass media advertising	23.5	-0.64***	0.09	1.01
	Social media influencer		-0.69***	0.08	0.09
	Family members or friends with prior experience using the product		0.16**	0.07	0.47
	Healthcare professional		1.17***	0.09	0.90
Effectiveness	Reduction of cardiovascular risk by 5%	15.7	-0.63***	0.08	0.87
	Reduction of cardiovascular risk by 10%		-0.07	0.07	0.27
	Reduction of cardiovascular risk by 15%		0.15**	0.06	0.22
	Reduction of cardiovascular risk by 20%		0.58***	0.07	0.80
Safety	No side effects	14.8	0.55***	0.06	0.70
	Mild side effects		0.72	0.05	0.19
	Moderate effects		-0.62***	0.07	0.67
Cost	250 THB/month (US$7.56)	8.1	0.28***	0.07	0.73
	500 THB/month (US$15.13)		0.26***	0.07	0.09
	1000 THB/month (US$30.26)		-0.16**	0.07	0.25
	1500 THB/month (US$45.39)		-0.38***	0.07	0.68
Opt-out			-0.57***	0.18	4.17

Note. All attributes were effects coded. Coefficients and standard errors for the reference levels were derived using the delta method based on the estimated covariance matrix (VARB). Statistical significance was assessed using Wald z-tests derived from the estimated coefficient and standard error.

The average exchange rate in 2025 was $US1 = 33.0453 THB [25]

AIC/N = 1.534; Log likelihood function = -4068.42.

***p-value < 0.01

**p-value < 0.05

Table S4 Comparison of parameters, Log-likelihood, McFadden R^2 and AIC/N between MNL, Full RPL and Reduced RPL models

Model	Parameters	Log-likelihood	McFadden R^2	AIC/N
MNL	14	-3929.90	-	1.796
Full RPL	28	-3414.61	0.292	1.568
Reduced RPL	24	-3839.88	0.204	1.760
Full RPL + sex interactions	42	-3407.07	0.294	1.571
Full RPL + HCP interactions	42	-3398.71	0.296	1.567
Full RPL + age ≥40 years interactions	42	-3389.58	0.298	1.563

HCP, Healthcare professional; MNL, The Multinomial Logit (MNL) Model

Table S5 Comparison of latent class logit (LCL) models between 2, 3, and 4 classes

Model	Log-likelihood	McFadden R^2	AIC/N	Largest class	Smallest class
2-class	-3496.68	0.275	1.606	60.9%	39.1%
3-class	-3422.71	0.291	1.579	39.3%	27.4%
4-class	-3376.69	0.300	1.565	38.5%	5.1%

Table S6 Comparison of 3 classes latent class logit (LCL) model with covariates

Model	Membership variables	Log-likelihood	AIC/N
3-class (no covariates)	None	-3422.71	1.579
3-class (8 variables)	Sex, being an HCP, income ≥ 30,000 THB, age ≥ 40 years, holding a bachelor’s degree or above, never use nutraceuticals, CVD preventive medications*, no CVD risk	-3398.96	1.575
3-class (4 variables)	Being an HCP, age ≥ 40 years, holding a bachelor’s degree or above, never use nutraceuticals	-3405.30	1.574

Note. *Cardiovascular preventive medications include antihypertensive agents, lipid-lowering medications, or antiplatelet agents

CVD, Cardiovascular disease

HCP, Healthcare professional

THB, Thai baht
